# Optimal approaches and criteria to treat-and-extend regimen implementation for Neovascular age-related macular degeneration: experts consensus in Taiwan

**DOI:** 10.1186/s12886-021-02231-8

**Published:** 2022-01-15

**Authors:** Cheng-Kuo Cheng, Shih-Jen Chen, Jiann-Torng Chen, Lee-Jen Chen, San-Ni Chen, Wen-Lu Chen, Sheng-Min Hsu, Chien-Hsiung Lai, Shwu-Jiuan Sheu, Pei-Chang Wu, Wei-Chi Wu, Wen-Chuan Wu, Chung-May Yang, Ling Yeung, Ta-Ching Chen, Chang-Hao Yang

**Affiliations:** 1 Department of Ophthalmology, Shin-Kong Wu Ho-Su Memorial Hospital, Taipei, Taiwan; 2 Department of Ophthalmology, School of Medicine, National Taiwan University, Taipei, Taiwan; 3 Department of Ophthalmology, School of Medicine, Fu-Jen Catholic University, New Taipei, Taiwan; 4 Department of Ophthalmology, School of Medicine, National Yang-Ming University, Taipei, Taiwan; 5 Department of Ophthalmology, Taipei Veterans General Hospital, Taipei, Taiwan; 6 Department of Ophthalmology, Tri-Service General Hospital, National Defense Medical Center, Taipei, Taiwan; 7 Department of Ophthalmology, Mackay Memorial Hospital, Taipei, Taiwan; 8 Department of Ophthalmology, Changhua Christian Hospital, Changhua, Taiwan; 9 Department of Ophthalmology, China Medical University Hospital, Taichung, Taiwan; 10 Department of Ophthalmology, College of Medicine, National Cheng Kung University, Tainan, Taiwan; 11 Department of Ophthalmology, National Cheng Kung University Hospital, College of Medicine, National Cheng Kung University, Tainan, Taiwan; 12 College of Medicine, Chang Gung University, Taoyuan, Taiwan; 13 Department of Ophthalmology, Chang Gung Memorial Hospital, Chiayi, Taiwan; 14 Department of Nursing, Chang Gung University of Science and Technology, Chiayi, Taiwan; 15 Department of Ophthalmology, Kaohsiung Medical University Chung-Ho Memorial Hospital, Kaohsiung, Taiwan; 16 Department of Ophthalmology, School of Medicine, Kaohsiung Medical University, Kaohsiung, Taiwan; 17 Department of Ophthalmology, Chang Gung Memorial Hospital- Kaohsiung Medical Center, Chang Gung University College of Medicine, Kaohsiung, Taiwan; 18 Department of Ophthalmology, Chang Gung Memorial Hospital-Linkou Medical Center, Chang Gung University College of Medicine, Taoyuan, Taiwan; 19 Department of Ophthalmology, National Taiwan University Hospital, No.8, Chung Shan S. Rd. (Zhongshan S. Rd.), Zhongzheng Dist., Taipei, 100226 Taiwan; 20 Department of Ophthalmology, Chang Gung Memorial Hospital, Keelung, Taiwan

**Keywords:** Anti-vascular endothelial growth factors, Expert opinion, Neovascular age-related macular degeneration, Treat-and-extend

## Abstract

The management of neovascular age-related macular degeneration (nAMD) has taken a major stride forward with the advent of anti-VEGF agents. The treat-and-extend (T&E) approach is a refined management strategy, tailoring to the individual patient’s disease course and treatment outcome. To provide guidance to implementing anti-VEGF T&E regimens for nAMD in resource-limited health care systems, an advisory board was held to discuss and generate expert consensus, based on local and international guidelines, current evidence, as well as local experience and reimbursement policies. In the experts’ opinion, treatment of nAMD should aim to maximize and maintain visual acuity benefits while minimizing treatment burden. Based on current evidence, treatment could be initiated with 3 consecutive monthly injections. After the initial period, treatment interval may be extended by 2 or 4 weeks each time for the qualified patients (i.e. no BCVA loss ≥5 ETDRS letters and dry retina), and a maximum interval of 16 weeks is permitted. For patients meeting the shortening criteria (i.e. any increased fluid with BCVA loss ≥5 ETDRS letters, or presence of new macular hemorrhage or new neovascularization), the treatment interval should be reduced by 2 or 4 weeks each time, with a minimal interval of 4 weeks. Discontinuation of anti-VEGF may be considered for those who have received 2–3 consecutive injections spaced 16 weeks apart and present with stable disease. For these individuals, regular monitoring (e.g. 3–4 months) is recommended and monthly injections should be reinstated upon signs of disease recurrence.

## Background

Age-related macular degeneration (AMD) is a complex and progressive ocular disease with poorly known underlying etiology, which could lead to irreversible central visual impairment or blindness. Albeit less common, neovascular AMD (nAMD) is an important subtype that accounts for over 90% of the severe vision loss in AMD patients [1]. Despite sharing similar clinical manifestations with nAMD, polypoidal choroidal vasculopathy (PCV) has a relatively more stable disease course and preferable long-term outcome [2]. PCV is typically featured by aberrant branching vascular network and aneurysmal dilation on indocyanine green angiography (ICGA) [3].

The prevalence of AMD was reported at 8.7% globally and 7.4% in Asians aging 45–85 years [4]. It has been projected that 288 million people worldwide would be affected by AMD in 2040, placing a palpable strain on both the health care system and the affected individuals [4]. Despite the fact that accurate estimation of PCV prevalence has largely been uneasy, the condition has been known to be substantially more common among Asians with presumed nAMD (22.3–54.7%), as compared with Caucasians (6–10%) [5–7]. In Taiwan, the prevalence of early and late AMD among adults aged 65 and above is estimated to be 15.0 and 7.3%, respectively, according to one cross-sectional study [8]. Since the prevalence of late AMD has been known to increase significantly with age, a rapidly aging society like Taiwan may expect to be confronted with severe disease burden and socioeconomic impacts [8].

With the advent of intravitreal anti-vascular endothelial growth factor (anti-VEGF) agents, the treatment goal of nAMD has shifted from salvaging vision to maintaining or improving visual outcomes while minimizing treatment burden [1, 9]. The anti-VEGF agents that have been used for ophthalmologic conditions include aflibercept, bevacizumab, and ranibizumab, and could be administered either by a reactive or proactive regimen [10]. However, as the need and response to anti-VEGF injections vary highly among nAMD patients, optimal, individualized regimens remain to be explored [11].

When a reactive, or pro re nata (PRN) approach is employed, injections are only given at the onset of symptomatic disease or signs of neovascular activity [11, 12]. While this individualized approach may reduce the number of injections when compared with monthly injections, regular monitoring is still warranted [13]. As shown in both trials and real world, PRN regimens generally lead to suboptimal visual outcomes in the absence of frequent monitoring and stringent retreatment criteria [9, 12]. Other notable drawbacks associated with the employment of PRN regimens in the clinical setting include delayed or under-treatment and logistical difficulties arising from the unpredictable dosing frequency [14].

Proactive approach, on the other hand, aims to minimize the risk of disease recurrence by giving regular injections at each scheduled visit, regardless of disease activity [10]. While fixed anti-VEGF dosing regimens have accumulated best trial evidence support, the implementation of this treatment approach in practice has been deemed impractical and could cause great burden to both the patient and health provider [12]. Treat-and-extend (T&E) regimen is a more flexible alternative to fixed dosing regimens, which involve a gradual lengthening of injection intervals upon the achievement of stable disease [15]. T&E regimen consists of initiation and extended maintenance phases [12]. During maintenance, regular injections are given at the scheduled visits and future treatment intervals are adjusted based on the patient’s functional and anatomic outcomes [10, 15]. Compared with fixed and PRN regimens, T&E regimens may have a number of important merits, including reducing the number of hospital visits, minimizing the risk of delayed, over- or under-treatment, and easing the logistical burden on the hospital and psychological stress on the patient [10].

In Taiwan, rigid National Health Insurance (NHI) policy and requirements have been imposed onto the prescription of anti-VEGF agents for ophthalmologic conditions. To be eligible for reimbursed anti-VEGF treatments for nAMD, patients have to be 50 years and above, and should have the diagnosis confirmed by fundus photography, fluorescence angiography (FA), and optical coherence tomography (OCT), and have a best-corrected visual acuity (BCVA) between 0.5 and 0.05. For the qualified patients, 8 intravitreal anti-VEGF injections would be granted with the first application, which are valid for 5 years. Patients with documented improvements may receive 3 doses with each subsequent application. Patients are entitled for a lifetime of 14 doses per eye and switching between two anti-VEGF agents is prohibited.

While the T&E approach appears to be an unequivocally appealing strategy for managing nAMD with intravitreal anti-VEGF, its implementation in a resource-limited health care system like Taiwan still warrants rigorous assessments and measured guidelines. Therefore, a panel of ophthalmology specialists were formed and met in Taipei on June 6th, 2020. The objective of this publication is to share the evidence-based recommendations gleaned from this meeting, which centered on the implementation of anti-VEGF T&E regimens in a resource-limited health care system. The facets of care addressed in this article pertain to the treatment goals of nAMD, initiation doses of an anti-VEGF therapy, the length of treatment interval extending/shortening, and the criteria for treatment adjustment and exit.

## Main text

### Methods

An expert panel consisting of fifteen local retina specialists was formed. In a face-to-face meeting, the group reviewed the literature on anti-VEGF T&E regimens in nAMD and worked toward developing consensus recommendations for the delivery of optimal nAMD care in Taiwan. The body of literature includes data from both prospective randomized controlled trials (RCTs) (Table 1) [13, 16–23] and real-world studies [24–29], as well as recommendations from the available guidelines [10, 14, 30]. Retrospective data on exit strategies following T&E regimens were also reviewed [31, 32]. A set of provisional statements and a management algorithm were formulated prior to the meeting, in accordance with current guideline recommendations, and reflecting the health care reimbursement policies and treatment pattern in Taiwan. The statements were examined and openly discussed, followed by anonymous voting. A consensus was considered to be reached when ≥70% experts voted in agreement. In the absence of consensus, further rounds of discussion, statement modification, and voting were involved until the acquisition of consensus.

**Table 1:** List of prospective RCTs that evaluated the employment of anti-VEGF T&E regimens in nAMD patients

Study Name	# of Eyes	Agent	Study Duration	Initial Dose	Extension / Shortening Interval	Min/Max Interval	VA Gain at 1 yr /2 yrs	# of Injection in 1 yr/ Through 2 yrs	% of Pts Achieved Treatment Internal ≥ 12 wks
TREND [13]	323	IVR	1 yr	Monthly injections until dry macula is achieved	+/− 2 wks	4/12 wks	6.2/n.a.	8.7/n.a.	22.3/n.a.
TREX-AMD [16]	40	IVR	2 yrs	3 monthly injections	+/− 2 wks	4/12 wks	10.4/8.7	10.1/18.6	n.a./n.a
CANTREAT [17]	287	IVR	2 yrs	3 monthly injections	+/− 2 wks	4/12 wks	8.4/6.8	9.4/17.6	n.a./n.a.
LUCAS [18, 19]	431	IVR	2 yrs	monthly injections until dry macula is achieved	+/− 2 wks	4/12 wks	8.7/7.4	8.0/16.0	17.0/n.a.
		IVB					8.4/6.6	8.9/18.2	10.0/n.a.
FLUID [20]	349	IVR	2 yrs	3 monthly injections	+/− 2 wks	4/12 wks	4.0/3.0	9.5/17.0	n.a.
							4.3/2.6	8.9/15.8	n.a.
RIVAL [21]	281	IVR	2 yrs	3 monthly injections	+/− 2 wks	4/12 wks	6.9/6.5	9.7/17.7	n.a.
		IVA					5.2/5.3	9.7/17.0	n.a.
ALTAIR [22]	255	IVA	2 yrs	3 monthly injections	+/− 2 wks +/− 4 wks	8/16 wks	9.0/7.6	7.2/10.4	56.8/56.9
							8.4/6.1	6.9/10.4	57.8/60.2
ARIES [23]	135	IVA	2 yrs	3 monthly injections	+/− 2 wks	8/16 wks	6.8/4.3	7.1/12.0	n.a./47.2

*IVA* Intravitreal aflibercept, *IVB* Intravitreal bevacizumab, *IVR* Intravitreal ranibizumab, *n.a.* Not available, *nAMD* Neovascular age-related macular degeneration, *RCT* Randomized controlled trial, *T&E* Treat-and-extend, *VA* Visual acuity, *VEGF* Vascular endothelial growth factor, *wk.* Week, *yr* Year

### Expert recommendations

**Fig. 1:**
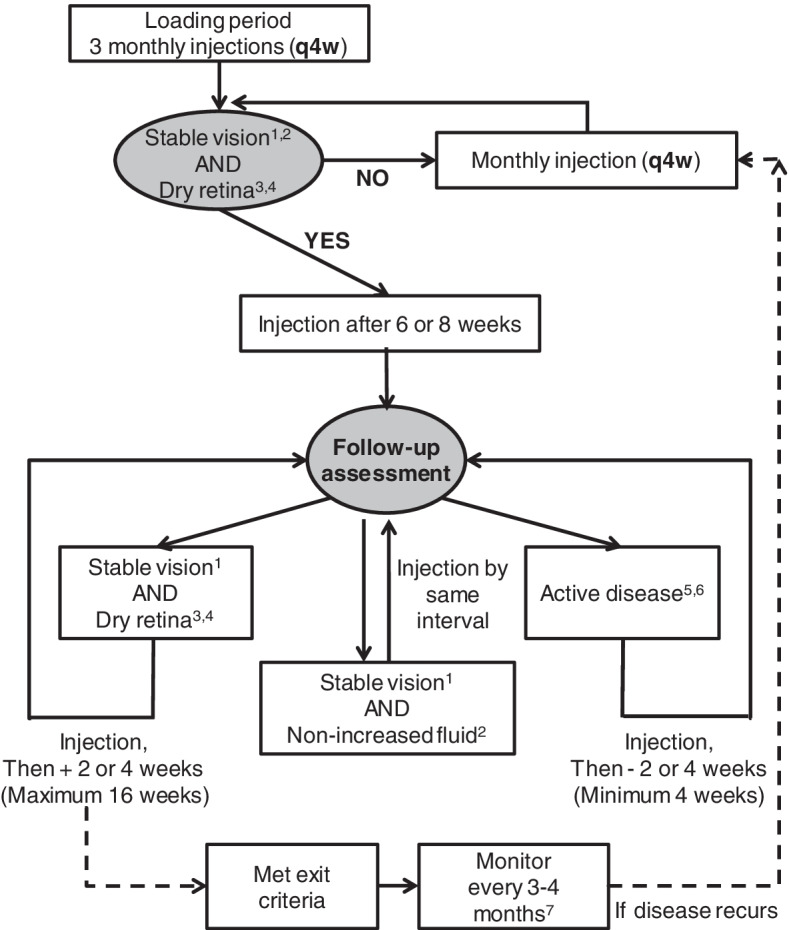
Management algorithm for nAMD patients undergoing anti-VEGF T&E regimens. ^1^Stable vision is defined as BCVA gain or BCVA loss < 5 ETDRS letters (or 1 line of Snellen chart). ^2^VA and OCT assessment should be conducted at visit of the third injection. ^3^Absence of macular hemorrhage and neovascularization is required. ^4^Non-increased fluid after 3 more consecutive monthly injections following initial treatment could be considered as persistent fluid, and the injection interval could be extended if VA is stable. ^5^Active disease is defined as any increased fluid with BCVA loss ≥5 ETDRS letters (or 1 line of Snellen chart), new macular hemorrhage, or new neovascularization. ^6^For patients with either increased fluid or BCVA loss ≥5 ETDRS letters alone, the treatment interval could be maintained or shortened. ^7^Patients who have met the exit criteria with serous PED should be monitored frequently (e.g. monthly or bi-monthly). BCVA, best-corrected visual acuity; ETDRS, Early Treatment Diabetic Retinopathy Study; nAMD, neovascular age-related macular degeneration; OCT, optical coherence tomography; PED, pigment epithelial detachment; T&E, treat-and-extend; VA: visual acuity; VEGF, vascular endothelial growth factor

The consensus recommendations and proposed management algorithm for the treatment of nAMD patients with anti-VEGF T&E regimens are exhibited in Table 2 and Fig. 1, respectively. The figure illustrates the general scheme of T&E regimens that may be adopted in the clinical setting. Following three consecutive injections at a 4-weekly interval (i.e. the initiation phase), treatment interval may be determined and adjusted based on the extension/shortening criteria at each visit.

**Table 2:** Recommendations of anti-VEGF T&E regimens for the management of nAMD

**Treatment goal** • The treatment goal of nAMD is to maximize and maintain VA benefits for patients while minimizing treatment burden.	
**Initiation of an anti-VEGF therapy** • Treatment could start with 3 consecutive monthly (or 4-weekly) injections.	
**Length of treatment interval extension/shortening** • After the initial treatment, patients meeting the extension criteria can have their treatment interval extended by 2 or 4 weeks at a time, with a maximum interval of 16 weeks. • For patients meeting the shortening criteria, the treatment interval should be reduced by 2 or 4 weeks at a time, with a minimal interval of 4 weeks.	
**Adjustment criteria** • Extension: No BCVA loss ≥5 ETDRS letters (or 1 line of Snellen chart) AND dry retina^a,b^ • Maintenance: No BCVA loss ≥5 ETDRS letters (or 1 line of Snellen chart) AND non-increased fluid^a^ • Shortening: Any increased fluid with BCVA loss ≥5 ETDRS letters (or 1 line of Snellen chart)^c^ OR new macular hemorrhage OR new neovascularization	
**Exit criteria** • Patients who have received 2–3 consecutive injections of 16 weeks apart with stable disease could consider exiting anti-VEGF treatment. • Patients exited from the anti-VEGF treatment should be followed every 3–4 months.^d^ Treatment regimen should be re-started from monthly dosing if disease recurs.	

^a^Absence of macular hemorrhage and neovascularization is required

^b^Non-increased fluid after 3 more consecutive monthly injections following initial treatment could be considered as persistent fluid, and the injection interval could be extended if VA is stable

^c^For patients with either increased fluid or BCVA loss ≥5 ETDRS letters alone, the treatment interval could be maintained or shortened

^d^Patients who have met the exit criteria with serous PED should be monitored frequently (e.g. monthly or bi-monthly)

*BCVA* Best-corrected visual acuity, *ETDRS* Early Treatment Diabetic Retinopathy Study, *nAMD* Neovascular age-related macular degeneration, *PED* Pigment epithelial detachment, *VA* Visual acuity, *VEGF* Vascular endothelial growth factor

#### Treatment goal

**Table:** 

• The treatment goal of nAMD is to maximize and maintain VA benefits for patients while minimizing treatment burden.	

In a published consensus document for the management of macular diseases, ophthalmic experts from the Vision Academy suggested that the treatment goal with anti-VEGF agents should aim to achieve and maintain BCVA, and treatment intervals should be adjusted to accommodate the patient’s needs [10]. In one consensus article published by a group of Taiwanese experts in 2020, the primary treatment goal for PCV was also the achievement of BCVA while minimizing patients’ treatment burdens [33]. In addition, one study from Japan reported that patients’ top expectations for intravitreal anti-VEGF regimens were reduced number of injections and maintenance of long-term VA [34]. Therefore, the experts uniformly agreed that nAMD treatments should aim to maximize BCVA while minimizing patients’ treatment burden.

#### Initiation of an anti-VEGF therapy

**Table:** 

• Treatment could start with 3 consecutive monthly (or 4-weekly) injections.	

Three consecutive monthly injections have been the frequently chosen dosing in most T&E RCTs as a means of anti-VEGF initiation, save for the TREND and LUCAS studies [13, 16–18, 20–23]. Take the ALTAIR study as an example, following the initiation phase that consisted of three monthly injections, trial participants were randomized to undergo T&E regimens with either a 2-week or 4-week adjustment. Maintenance of treatment interval was also permitted when the adjustment criteria were not met [22]. The BCVA improvement peaked after the three initial doses and maintained thereafter. At week 52, the mean change in BCVA from baseline was 9.0 and 8.4 letters in the 2-week group and 4-week group, respectively. At week 96, about 60% of patients in both treatment arms maintained at least a 12-week injection interval [22]. Therefore, three monthly injections may indeed be a good choice for anti-VEGF therapy initiation.

For the treatment of nAMD with aflibercept, both Asia-pacific and UK experts recommended giving three monthly injections as the initial dose [14, 30]. The three monthly injections have also been a commonly recommended initial dose for PCV. For example, in one consensus report on PCV management, Taiwanese experts also recommended initiating anti-VEGF treatments with three monthly injections [33]. As a result, the experts agreed that three consecutive monthly injections could be an appropriate initial dosing option for anti-VEGF T&E regimens. However, a few studies have suggested that comparable outcomes could be achieved with just one injection [35, 36]. Hence, certain flexibility could be reserved for the dosing of anti-VEGF in the initiation phase, to accommodate for the multitude of factors in the real-world clinical practice.

For those who show no signs of improvement after numerous monthly injections, the possibility of other ophthalmic conditions should be reconsidered and relevant examinations such as OCT, color fundus examination, FA, and ICGA may be re-evaluated [33]. If other diagnoses are suspected, guidelines should be consulted for further diagnostic and management details.

#### Length of treatment interval extension/shortening

**Table:** 

• After the initial period of an anti-VEGF therapy, patients meeting the extension criteria can have their treatment interval extended by 2 or 4 weeks at a time, with a maximum interval of 16 weeks. • For patients meeting the shortening criteria, the treatment interval should be reduced by 2 or 4 weeks at a time, with a minimal interval of 4 weeks.	

In the published T&E trials, the frequently used minimal and maximum treatment intervals have been 4 or 8 weeks and 12 or 16 weeks, respectively [13, 16–23]. While the treatment intervals of these trials have mainly been extended or shortened by a 2-week increment, a 4-week increment was allowed in the ALTAIR study. Having considered both the literature evidence and practicality, the experts decided that the treatment interval should be adjusted by 2 or 4 weeks at a time, with a minimal interval of 4 weeks and a maximal interval of 16 weeks.

#### Treatment adjustment criteria

**Table:** 

• Extension: No BCVA loss ≥5 Early Treatment Diabetic Retinopathy Study (ETDRS) letters (or 1 line of Snellen chart) AND dry retina^§,†^ • Maintenance: No BCVA loss ≥5 ETDRS letters (or 1 line of Snellen chart) AND non-increased fluid^§^ • Shortening: Any increased fluid with BCVA loss ≥5 ETDRS letters (or 1 line of Snellen chart)^‡^ OR new macular hemorrhage OR new neovascularization ^§^Absence of macular hemorrhage and neovascularization is required. ^†^Non-increased fluid after 3 more consecutive monthly injections following initial treatment could be considered as persistent fluid, and the injection interval could be extended if VA is stable. ^‡^For patients with either increased fluid or BCVA loss ≥5 ETDRS letters alone, the treatment interval could be maintained or shortened.	

While adjustment has been the core of the T&E regimens, the experts pointed out that the option of maintenance is also of importance and should be reserved in the clinical setting for practicality reasons (e.g. avoiding frequent adjustments, especially for patients presented with stable vision and without increased fluid). The experts formulated a set of sufficient conditions for treatment interval adjustment based on the T&E design of the relevant trials [13, 17–19, 22, 23]. Stable vision and the absence of macular hemorrhage and neovascularization are the necessary conditions for treatment interval extension or maintenance. Based on clinical observation, full resolution of retinal fluid may never occur in some patients despite continued treatment. Therefore, residual fluid after the three initial monthly injections may be considered as persistent fluid. For patients with stabilized VA and persistent fluid, treatment extension may be considered.

Correlations between the volume of specific fluid compartments and BCVA outcomes have been described in previous studies [37, 38]. By utilizing AI technology to analyze the optical coherence tomography (OCT) data and quantify the volumes of intraretinal fluid (IRF), subretinal fluid (SRF), and pigment epithelial detachment (PED), Schmidt-Erfurth et al. also demonstrated an association between increased IRF and BCVA regression, whereas increased SRF appears to be a weak positive prognostic factor for BCVA [39]. While doing AI-analysis of another study with smaller number of enrollment, the same research team recently identified a spatial relationship between SRF and vision outcomes, i.e. SRF in the juxtafoveal area may slightly impact vision in a volume-dependent manner, but SRF in the central fovea had neutral effect on VA. On the other hand, IRF in the central fovea was associated with worse VA, whereas IRF in the juxtafovea area has no significant correlation to VA outcome [40]. The slight discrepancy between these two studies could be due to the better baseline vision acuity and smaller total fluid volume to be analyzed in the more recent study [40]. Based on these findings, AI technology may potentially be employed in the future to predict treatment outcomes through the precise determination of the fluid type, volume, and location. However, given that consensus on the management approach for each of the fluids was not reached among the experts, the treatment recommendation herein is not specified by fluid type.

For patients presenting with either retinal fluid increase or VA regression alone, the decision to maintain or shorten the treatment interval may be left at the physician’s discretion, as a number of factors may need to be considered. For example, in cases with increased fluid alone, choroidal neovascularization (CNV) may recur prior to the onset of VA decrease. Therefore, the component of the fluid is of relevance. If IRF was the predominant fluid type, the injection interval should be shortened. If SRF was the main source of the increased fluid, the treatment decision would then be determined by the level of the increase. However, the criterion of SRF thickness for treatment adjustment has varied across trials. For example, treatment intervals were shortened at the presence of SRF of ≥50 um in ARIES, ≥100 um central retina thickness (CRT) in ALTAIR, and ≥ 200 um in FLUID. For patients with only BCVA loss greater than 5 ETDRS, supplementary FA or fundus autofluorescence (FAF) may be performed to exclude fovea atrophy or other vitreomacular interface problems.

#### Treatment exit criteria

**Table:** 

• Patients who have received 2–3 consecutive injections of 16 weeks apart with stable disease could consider exiting anti-VEGF treatment. • Patients exited from the anti-VEGF treatment should be followed every 3–4 months.^¶^ Treatment regimen should be re-started from monthly dosing if disease recurs. ^¶^Patients who have met the exit criteria with serous PED should be monitored frequently (e.g. monthly or bi-monthly).	

The exit criteria for anti-VEGF treatment in nAMD patients vary across published studies. In 2017, Adrean et al. reported that nAMD patients may successfully stop anti-VEGF treatment while maintaining improved vision after the completion of a Treat-Extend-Stop protocol, where treatment may be stopped for those maintaining stable disease after two consecutive 12-weekly injections [31]. In a study that involved patients managed with a T&E protocol, Arendt et al. defined the exit criteria as three consecutive injections at a 16-week interval with stable disease [32]. In a consensus that addressed various aspects of nAMD treatment with aflibercept, a group of Asia-Pacific experts suggested that treatment cessation may be considered for stabilized patients who have received injections at 12-week intervals for 1 year [30]. British experts also recommended that patients with stable disease after three consecutive injections 16 weeks apart may be considered for treatment cessation [14]. Based on these evidences, the experts decided that flexibility should be reserved for treatment cessation decisions, and two to three consecutive injections 16 weeks apart with stable disease may be a practical exit criterion.

After exiting from treatment, patients should be regularly followed every 3–4 months to monitor for disease recurrence. Of note, a more frequent monitoring is warranted for patients exiting treatment with serous PED, as the risk of recurrence has been reported to be higher among these patient [32]. To facilitate the delivery of timely intervention during the follow-up period, patients should also receive adequate education on the means of self-monitoring and the early signs of disease recurrence. Monthly injections should be resumed upon recurrence and treatment intervals may be gradually extended thereafter.

## Conclusion

Efficacious treatments that offer improvements in and maintenance of VA gains are of paramount importance to patients with nAMD [10]. The T&E strategy has been forged with the intention to improve disease control and the patient’s quality of life and to reduce treatment burden. Based on the relevant trial data and currently available guideline recommendations, this paper examined the practical aspects pertinent to implementing a suitable T&E strategy for nAMD patients receiving anti-VEGF injections. The recommendations were formed to provide guidance to ophthalmologists practicing in a resource limited health care system. Amid the raging pandemic of COVID-19, while the fixed dose strategy has been recommended for managing retinal diseases to minimize exposure of patients and healthcare personnel to COVID-19 [41], Taiwanese experts felt that whenever the situation permits, treatment intervals should be tailored to conform to patients’ will, hospital capacity, and the local public health policies.

## Data Availability

Data sharing is not applicable to this article as no datasets were generated or analyzed during the current study.

## Abbreviations

BCVA: Best-corrected visual acuity
CNV: Choroidal neovascularization
CRT: Central retina thickness
ETDRS: Early Treatment Diabetic Retinopathy Study
FA: Fluorescence angiography
FAF: Fundus autofluorescence
ICGA: Indocyanine green angiography
IRF: Intraretinal fluid
nAMD: Neovascular age-related macular degeneration
NHI: National Health Insurance
OCT: Optical coherence tomography
PCV: Polypoidal choroidal vasculopathy
PED: Pigment epithelial detachment
PRN: Pro re nata
RCT: Randomized controlled trial
SRF: Subretinal fluid
T&E: Treat-and-extend
VA: Visual acuity
VEGF: Vascular endothelial growth factors
